# Supplementary material for: Measuring Exciton Fine-Structure in Randomly Oriented Perovskite Nanocrystal Ensembles Using Nonlinear Optical Spectroscopy: Theory
Source: Nanomaterials (Basel). 2022 Feb 26;12(5):801. doi: 10.3390/nano12050801 (PMC8912615; doi:10.3390/nano12050801)
Supplement: Supplementary file 1 [file nanomaterials-12-00801-s001.zip › nanomaterials-1608939-supplementary.pdf]

Supporting Information for:
Measuring Exciton Fine-Structure in Randomly Oriented Perovskite Nanocrystal Ensembles Using Nonlinear Optical Spectroscopy: Theory

Contents

Enumerating Quantum Pathways Using Feynman Diagrams 1
Constructing Feynman Diagrams 1
Linear Optical Response 2
Third-Order Optical Response 2

Analytical Form of the Optical Response Functions 3

Theory of Rotational-Averaging 4

References 5

ENUMERATING QUANTUM PATHWAYS USING FEYNMAN DIAGRAMS

An nth-order nonlinear optical process consists of $n+1$ field-matter interactions. In such a perturbative picture, the response function is determined by a sequence of n nested commutators:

$$S^{(n)}(t_n, t_{n-1}, \dots t_1) \propto \mathrm{Tr}\left[\mu(t_n + t_{n-1} + \dots t_1), [\mu(t_{n-1} + t_{n-2} + \dots t_1), [\dots[\mu(0), \rho(t=0)]\dots]]\right] \tag{S1}$$

where $\mu(t) = e^{+\frac{i}{\hbar}H_0 t}\mu e^{-\frac{i}{\hbar}H_0 t}$ is the transition dipole operator in the interaction picture and H_0 is the unperturbed system Hamiltonian. The argument of the trace operation may be intuitively thought of as propagation of the initial density matrix $\rho(t=0)$, which lends itself to a diagrammatic representation as discussed below. The nonlinear polarization in response to an applied field $E(t)$ is then:

$$P^{(n)}(t) = \int_0^\infty \int_0^\infty \cdots \int_0^\infty S^{(n)}(t_n, t_{n-1}, \dots t_1) E(t-t_3) E(t-t_3-t_2) \dots E(t-t_3-t_2 \cdots - t_1) dt_1 \dots dt_{n-1} dt_n \tag{S2}$$

It can be shown straightforwardly that for impulsive excitation by delta function pulses, each polarization term $P^{(n)}$ becomes identical to its respective optical response function $S^{(n)}$ with the appropriate time-arguments.

The n nested commutators in equation (S1) result in multiple distinct terms, or quantum pathways, which comprise the nth-order optical response function. However, the number and complexity of these quantum pathways increases with rapidly with n and the number of resonant transitions. This motivates the use of so-called double-sided Feynman diagrams to enumerate quantum pathways in a given spectroscopic experiment.

Constructing Feynman Diagrams

We begin by briefly defining terminology. Each spectroscopic technique that probes an nth-order response function may be reduced to measurement of a signal emitted in a phase-matching direction $\mathbf{k}_{\mathrm{sig}} = \pm\mathbf{k}_1 \pm \mathbf{k}_2 \cdots \pm \mathbf{k}_n$, where $\mathbf{k}_i$ is the wavevector of each respective excitation field. The (negative)positive sign preceding each wavevector in the equation for $\mathbf{k}_{\mathrm{sig}}$ denotes each field as (non-)conjugated.

The rules for constructing Feynman diagrams for all possible quantum pathways are then simply:

1. Time runs vertically upwards, with the initial system density matrix $\rho(t=0)$ at the bottom.
2. Each field-matter interaction is represented by an arrow which points (right)left for a (non-)conjugated field.
3. Arrows may be located either on the right or left side of the density matrix elements, which effect a change in either the Bra or Ket respectively. An arrow pointing (out)inward corresponds to (de-)excitation of the element. Diagrams that break this rule vanish via the rotating-wave approximation.

4. The sign of each diagram is given by $(-1)^m$, where m is the number of interactions involving the Bra element on the right side.

5. Each diagram must end in a population state.

We now demonstrate examples of linear and third-order Feynman diagrams constructed from these rules.

Linear Optical Response

Consider the level system in Figure S1a consisting of ground and excited states $|g\rangle$ and $|e\rangle$ as well as a doubly-excited state $|d\rangle$. In anticipation of two-photon absorption, we also include an intermediate virtual state $|v\rangle$ in the middle of the energy gap. For weak excitation fields, the linear polarization $P^{(1)}(t)$ (underlying one-photon absorption and fluorescence) dominates the optical response. From a time-domain perspective, one may begin from interaction of the system with a single excitation laser pulse as shown in Figure S1b. Subsequent optical absorption is represented by the Feynman diagram in Figure S2c, in which the light-matter converts the initial equilibrium ground state $|g\rangle\langle g|$ into a coherence $|e\rangle\langle g|$. After propagating for a time t, a second interaction with the pulse converts this coherence into a final excited population state $|e\rangle\langle e|$. Fourier transforming the coherence dynamics along t then generates the usual absorption spectrum. The Feynman diagram representing fluorescence is then shown in Figure S1d, which is simply the inverse process of absorption. As there is no excitation field however, the physical origin of the light-matter interaction in fluorescence may, in the current semi-classical picture, be informally connected to perturbation by the vacuum field.

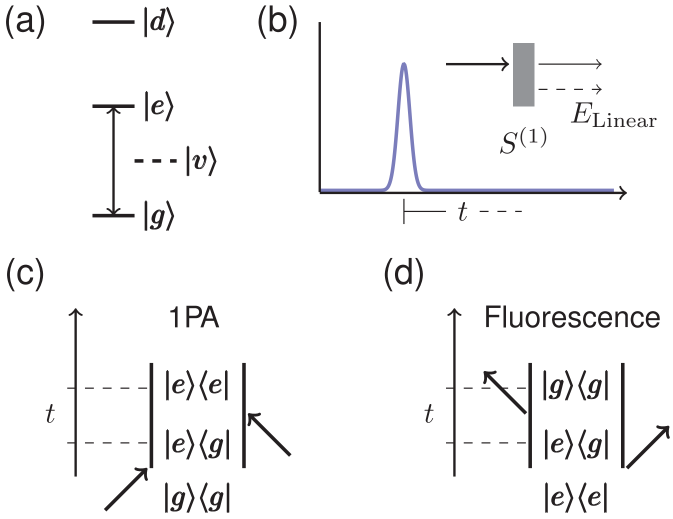

Figure S1: (a) Three-level system consisting of a ground ($|g\rangle$), singly-excited ($|e\rangle$), and doubly-excited ($|d\rangle$) state. A mid-gap virtual state ($|v\rangle$) is also shown which is accessed in two-photon absorption. (b) Single pulse excitation of a material which generates a time-dependent linear optical response. (c,d) Feynman diagrams of linear (c) one-photon absorption and (d) fluorescence.

Third-Order Optical Response

We now examine the third-order optical response which generates a four-wave mixing signal. Before the general case of three excitation pulses in Figure S2a we consider two-photon absorption, a third-order analogue to linear one-photon absorption. The Feynman diagram representing two-photon absorption is shown in Figure S2b, which consists of four excitation processes that traverse an intermediate virtual state population $|v\rangle\langle v|$ and culminate in a final excited state population $|e\rangle\langle e|$. This Feynman diagram representation may be related to the traditional transition diagrams used to visualize the process [1]. Next, the ground state bleach (GSB), excited state emission (ESE), and excited state absorption (ESA) diagrams discussed in the main text are plotted in Figure S2c-d respectively for the pulse sequence shown in Figure S2a. Note that according to rule 4 stated above, the GSB and ESE diagrams have positive signs while the ESA diagram has a negative sign, which reflects its absorptive nature.

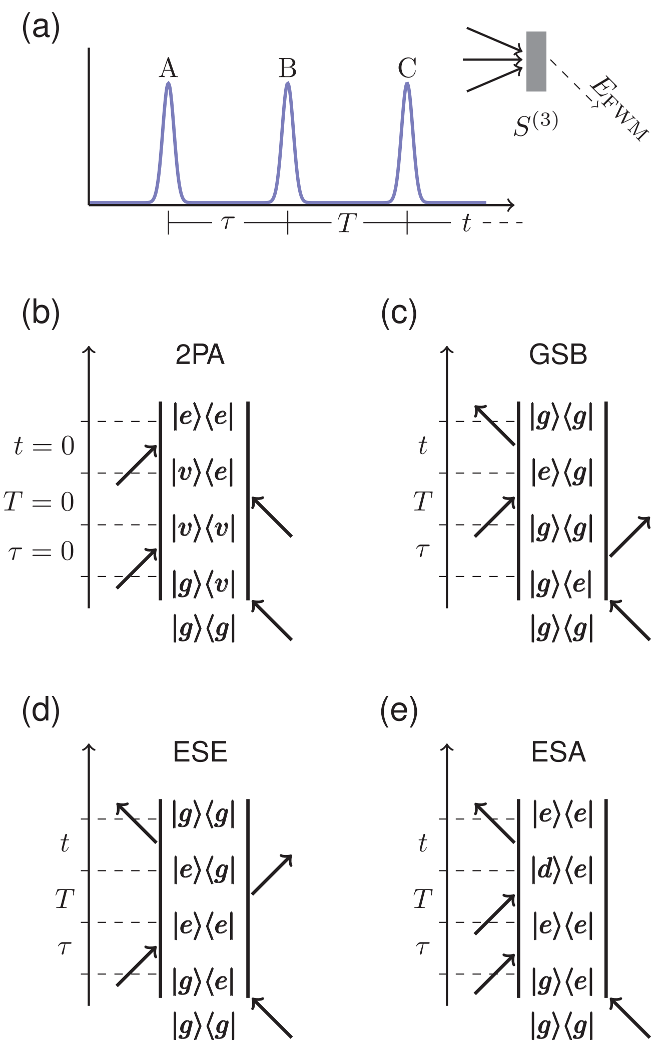

Figure S2: (a) Three-pulse excitation sequence that generates a four-wave mixing signal. (b-e) Feynman diagrams representing (b) two-photon absorption, (c) ground state bleach, (d) excited state emission, and (e) excited state absorption processes.

ANALYTICAL FORM OF THE OPTICAL RESPONSE FUNCTIONS

The Feynman diagrams shown above may be easily translated to analytical expressions for their corresponding optical response functions. We consider only the ESE diagram here, but the expressions for the GSB and ESA diagrams may be obtained in an identical fashion and are described elsewhere [2, 3].

The ESE diagram is reproduced in Figure S3 along with its corresponding contribution to the optical response function. Each time delay is highlighted in a specific color, and gives rise to specific components of the response function $R^{(3)}\ _{\text{ESE}}$ which are highlighted in matching colors:

1. Time delay τ (red): The dynamics along delay τ are of the density matrix element $|g\rangle\langle e|$, which corresponds to coherence oscillation of the form $e^{-i\omega_{eg}\tau}$. Assuming Markovian dephasing [3], the coherence will also experience dephasing of the form $e^{-i\Gamma_{eg}\tau}$, where Γ_{eg} is the dephasing rate.

2. Time delay T (green): The dynamics along delay T are of the density matrix element $|e\rangle\langle e|$, which corresponds to population relaxation of the form $e^{-i\Gamma_{ee}T}$, where Γ_{ee} is the population relaxation rate. Note that in the case of additional ground or excited state levels, intraband coherence oscillation may also occur along this time delay.

3. Time delay t (blue): The dynamics along delay t are of the density matrix element $|e\rangle\langle g|$, which corresponds to coherence oscillation of the form $e^{+i\omega_{eg}\tau}$. Note that due to the mirror density matrix element the coherence

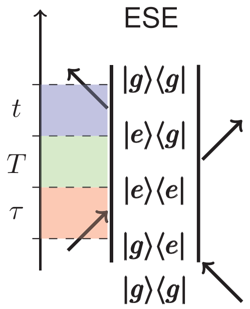

$$R^{(3)}_{\mathrm{ESE}} = \left(\frac{i}{\hbar}\right)^3 \left[e^{-i\omega_{eg}\tau} e^{+i\omega_{eg}t} e^{-\Gamma_{eg}\tau} e^{-\Gamma_{ee}T} e^{-\Gamma_{eg}t}\right]$$

Figure S3: Excited state emission Feynman diagram and corresponding analytical expression for its contribution to the optical response function. The highlighted colors connect each component of the optical response function to its corresponding time delay, along which the dynamics evolve.

oscillation is of opposite sign. Again, dephasing occurs of the form $e^{-i\Gamma_{eg}t}$, where Γ_{eg} is the dephasing rate.

THEORY OF ROTATIONAL-AVERAGING

In the notation of Andrews and Thirunamachandran [4], a general spectroscopic observable may be written in the form:

$$C = \sum_{i_1 \ldots i_n} A_{i_1 \ldots i_n} P_{i_1 \ldots i_n} \tag{S3}$$

where $\mathrm{A}_{i_1 \ldots i_n}$ and $\mathrm{P}_{i_1 \ldots i_n}$ are nth-rank tensors representing the excitation polarization(s) and the transition strength(s) of a system. Both tensors are expressed in a space-fixed experimental reference frame indexed by i_m. Of course, the transition strength tensor of a nanocrystal is more naturally expressed in the nanocrystal reference frame and may be transformed as:

$$P_{i_1 \ldots i_n} = \sum_{\lambda_1 \ldots \lambda_n} L_{i_1 \lambda_1} \ldots L_{i_n \lambda_n} P_{\lambda_1 \ldots \lambda_n} \tag{S4}$$

where λ_m indexes the nanocrystal reference frame and $L_{i_m \lambda_m}$ is the direction cosine between the two axes i_m and λ_m. The optical response of an ensemble may thus be written:

$$\begin{aligned} \langle C \rangle &= \sum_{i_1 \ldots i_n, \lambda_1 \ldots \lambda_n} A_{i_1 \ldots i_n} \langle L_{i_1 \lambda_1} \ldots L_{i_n \lambda_n} \rangle P_{\lambda_1 \ldots \lambda_n} \\ &= \sum_{i_1 \ldots i_n, \lambda_1 \ldots \lambda_n} A_{i_1 \ldots i_n} I^{(n)}_{i_1 \ldots i_n, \lambda_1 \ldots \lambda_n} P_{\lambda_1 \ldots \lambda_n} \end{aligned} \tag{S5}$$

where the brackets $\langle \ldots \rangle$ denote a rotational average over all possible orientations and the elements of the coordinate interface tensor $I^{(n)}$ may be systematically evaluated [4]. Here we will consider linear and third-order spectroscopies, which require the elements of $I^{(2)}$ and $I^{(4)}$ respectively:

$$I^{(2)}_{i_1 i_2; \lambda_1 \lambda_2} = \frac{1}{3} \delta_{i_1 i_2} \delta_{\lambda_1 \lambda_2} \tag{S6}$$

$$\begin{aligned} & I^{(4)}_{i_1 i_2 i_3 i_4; \lambda_1 \lambda_2 \lambda_3 \lambda_4} \\ & = \frac{1}{30} \begin{pmatrix} \delta_{i_1 i_2} \delta_{i_3 i_4} \\ \delta_{i_1 i_3} \delta_{i_2 i_4} \\ \delta_{i_1 i_4} \delta_{i_2 i_3} \end{pmatrix}^{\mathrm{T}} \begin{pmatrix} 4 & -1 & -1 \\ -1 & 4 & -1 \\ -1 & -1 & 4 \end{pmatrix} \begin{pmatrix} \delta_{\lambda_1 \lambda_2} \delta_{\lambda_3 \lambda_4} \\ \delta_{\lambda_1 \lambda_3} \delta_{\lambda_2 \lambda_4} \\ \delta_{\lambda_1 \lambda_4} \delta_{\lambda_2 \lambda_3} \end{pmatrix} \end{aligned} \tag{S7}$$

We next construct the the polarization tensor $A_{i_1 \ldots i_n}$. Defining the propagation direction of our excitation beams as $\hat{z}$, we can write the possible linear and circular polarization vectors as follows:

$$\mathbf{p}_\mathrm{X} = \begin{pmatrix} 1 \\ 0 \\ 0 \end{pmatrix}, \quad \mathbf{p}_\mathrm{Y} = \begin{pmatrix} 0 \\ 1 \\ 0 \end{pmatrix} \tag{S8}$$

$$\mathbf{p}_\mathrm{R} = \frac{1}{\sqrt{2}} \begin{pmatrix} 1 \\ -i \\ 0 \end{pmatrix}, \quad \mathbf{p}_\mathrm{L} = \frac{1}{\sqrt{2}} \begin{pmatrix} 1 \\ +i \\ 0 \end{pmatrix} \tag{S9}$$

The polarization tensor $A_{i_1 \ldots i_n}$ is then constructed by the tensor product of n polarization vectors corresponding to n experimental light-matter interactions:

$$A_{i_1 \ldots i_n} = \mathbf{p}_{i_1} \otimes \mathbf{p}_{i_2} \cdots \otimes \mathbf{p}_{i_n} \tag{S10}$$

Likewise, the transition strength tensor is constructed by the tensor product of n transition moment vectors:

$$P_{\lambda_1 \ldots \lambda_n} = \boldsymbol{\mu}_{\lambda_1} \otimes \boldsymbol{\mu}_{\lambda_2} \cdots \otimes \boldsymbol{\mu}_{\lambda_n} \tag{S11}$$

Here, we take each $\boldsymbol{\mu}_{\lambda_m}$ to be a unit vector in order for $\langle C \rangle$ to act as a weighting factor ranging from zero to unity.

[1] D. M. Friedrich, J. Chem. Educ. **59**, 472 (1982), ISSN 0021-9584, URL `https://doi.org/10.1021/ed059p472`.

[2] P. Hamm and M. Zanni, *Concepts and Methods of 2D Infrared Spectroscopy* (Cambridge University Press, 2011), 1st ed.

[3] A. Liu and S. T. Cundiff, Phys. Rev. Mater. **4**, 055202 (2020), URL `https://link.aps.org/doi/10.1103/PhysRevMaterials.4.055202`.

[4] D. L. Andrews and T. Thirunamachandran, J. Chem. Phys. **67**, 5026 (1977), ISSN 0021-9606, URL `https://doi.org/10.1063/1.434725`.
